# Impact of Uterine Leiomyomas on Cardiovascular Disease Risk in Young Reproductive-Aged Women: A Nationwide Population-Based Cohort Study

**DOI:** 10.3390/jcm14020519

**Published:** 2025-01-15

**Authors:** Jung Yoon Park, Kyungdo Han, Hyunkyung Kim, Jae-Yen Song, Mee-Ran Kim, Youn-Jee Chung

**Affiliations:** 1 Division of Reproductive Endocrinology, Department of Obstetrics and Gynecology, Seoul St. Mary’s Hospital, College of Medicine, The Catholic University of Korea, 222 Banpo-daero, Seocho-gu, Seoul 06591, Republic of Korea; aurorix86@naver.com (J.Y.P.); jaeyen77@nate.com (J.-Y.S.); drmrkim@gmail.com (M.-R.K.); 2 Department of Statistics and Actuarial Science, Soongsil University, Seoul 06978, Republic of Korea; hks@ssu.ac.kr; 3 Department of Obstetrics and Gynecology, Uijeongbu St. Mary’s Hospital, College of Medicine, The Catholic University of Korea, Seoul 06591, Republic of Korea; hyuna17@catholic.ac.kr

**Keywords:** leiomyoma, cardiovascular disease, myocardial infarction, ischemic stroke, uterine myomectomy, reproduction, epidemiology

## Abstract

**Background/Objectives:** Uterine leiomyomas are the most common benign gynecological tumors in women of reproductive age and are often associated with localized symptoms. However, emerging evidence suggests a link between uterine leiomyomas and systemic conditions such as cardiovascular disease (CVD), particularly myocardial infarction (MI) and ischemic stroke (IS). This study aimed to investigate the relationship between uterine leiomyomas and the risk of CVD events in young women aged 20–39 years using a large, nationwide, population-based cohort. **Methods:** This retrospective cohort study analyzed data from the National Health Insurance Service database of South Korea of 2,581,700 women aged 20–39 years who underwent health screening between 2009 and 2012. Uterine leiomyomas were identified using International Code of Disease, 10th Edition codes. CVD events (MI and IS) were defined according to hospital claims and radiological data. Multivariate hazard ratios (HRs) were calculated to evaluate the association between leiomyomas and CVD after adjusting for confounders (age, lifestyle factors, comorbidities, and body mass index). **Results:** In all, 58,812 were diagnosed with uterine leiomyomas, and 25,063 underwent surgical treatment. During the follow-up period, MI occurred in 115 women (0.21%) and IS in 82 women (0.15%) in the leiomyoma group, compared with 3107 cases of MI (0.12%) and 2240 cases of IS (0.09%) in the non-leiomyoma group. The leiomyoma group demonstrated a higher incidence rate of CVD (IR: 0.63 vs. 0.39 per 1000 person-years). After adjusting for confounding factors such as age, lifestyle, comorbidities, and body mass index (BMI), the hazard ratio (HR) for MI was 1.32, indicating a statistically significant increase in cardiovascular risk. The risk of CVD was lower in women who underwent surgical treatment; however, when specifically analyzing the occurrence of MI and IS, no statistically significant differences were observed between the two groups. **Conclusions:** Uterine leiomyomas are associated with an increased risk of MI and IS in young women. Surgical treatment itself may be associated with additional cardiovascular risks. Further research is needed to develop strategies to mitigate these risks and elucidate the underlying mechanisms.

## 1. Introduction

Uterine leiomyomas, also known as myomas, are the most prevalent benign gynecological tumors arising from the uterine smooth muscle tissue, affecting up to 68.6% of women [1,2,3]. Over the past 30 years, the number of prevalent cases of uterine myomas worldwide has increased from 126.41 million to 226.05 million, marking a growth rate of 78.82%. Globally, the incidence and prevalence rates are reported to be on the rise among those aged 25–29, with the highest rates observed in the 35–39 age group [4]. Myomas can lead to heavy menstrual bleeding in women of reproductive age and may also cause pressure-related symptoms, such as bowel and bladder dysfunction, abdominal distension, pelvic pain, and infertility [5,6]. Although myomas are predominantly viewed as a gynecological condition, emerging evidence suggests they may have systemic implications, particularly regarding cardiovascular health [7,8,9,10]. Menorrhagia, a common symptom of myoma, is associated with iron deficiency anemia, which can impair oxygen delivery and negatively impact cardiovascular metabolic health [11,12]. An analysis of patients hospitalized due to menorrhagia in the United States revealed that among patients under 40 years old, those hospitalized for heavy menstrual bleeding exhibited a higher risk of cardiovascular disease [13]. Furthermore, national data indicate that anemia in young women is associated with an increased risk of acute myocardial infarction, stroke, cerebrovascular disease, and mortality a decade later, suggesting the need for heightened awareness of related risk factors in this population [14]. In the case of hypertension, a major risk factor for cardiovascular disease, the potential association with myomas has been suggested in several studies over the years, and meta-analyses have demonstrated a significant correlation; however, this relationship remains controversial [15,16,17,18,19,20].

Cardiovascular disease (CVD) is the leading cause of death among women in South Korea. According to the 2023 mortality statistics, the mortality rate due to cardiovascular disease was observed to be 6.6% higher in women (136.3 per 100,000 population) than in men (127.8 per 100,000 population) [21]. Over the past five years, the number of patients with heart disease has gradually increased by 19.9% compared to 2018 (from 1.52 million to 1.83 million). Notably, among women, there was a significant increase of 40.9% in their 20s and 27.3% in their 30s, indicating a marked rise in the prevalence of cardiovascular disease among younger patients under 40 [21]. This trend highlights the increasing importance of cardiovascular disease in younger populations. In light of the increasing prevalence of myomas and the rising trend of cardiovascular disease among young women, this study aims to investigate the impact of myomas on cardiovascular health in young women through a large, population-based retrospective cohort analysis.

## 2. Materials and Methods

### 2.1. Data Source

We used retrospective review data from the National Health Information Database of the National Health Insurance Service (NHIS), a public database on healthcare utilization, health screening, sociodemographic variables, and mortality for the entire population of South Korea. As the Korean NHIS is a universal coverage health insurance system, it was constructed to provide public health researchers and policymakers with useful, representative information regarding the utilization of health insurance and health examinations.

### 2.2. Study Population

Of a total 2,755,790 women aged 20–39 years who used the health check-up service of the NHIS from 1 January 2009 to 31 December 2012, we identified 2,598,187 eligible women, excluding individuals who had any missing data. We excluded individuals diagnosed with MI (*n* = 7526) or IS (*n* = 5592) before the health check-up date. Those who were diagnosed with uterine myoma, MI, or IS within the first 3 years after the inception of the database were excluded (*n* = 3369). A total of 2,581,700 individuals were included in this study. Individuals in the cohort were followed from the screening date to 31 December 2018.

### 2.3. Definitions of Events

Uterine myomas were diagnosed using standardized codes from the Korean version of the International Code of Disease, 10th Edition (ICD-10). The ICD code for uterine myoma was D25. We selected participants who claimed ≥1 time for hospitalization or 2 times for outpatient care or underwent surgical treatment for myomas.

The occurrence of MI was defined as an ICD-10 code I21 or I22, claimed at least twice or more than once during hospitalization. The occurrence of IS was defined as an ICD-10 code I63 or I64, claimed together with hospitalization and radiological examination (magnetic resonance imaging or computed tomography). Participants with a history of MI or IS identified using ICD code claims prior to the baseline were excluded.

### 2.4. Other Baseline Characteristics

Comorbidities such as hypertension, diabetes mellitus (DM), dyslipidemia, and chronic kidney disease (CKD) were defined using previous claims records of the ICD-10 codes. Sociobehavioral information such as smoking history, drinking (>30 g of alcohol per day), regular physical activity (moderate exercise for >3 days per week or vigorous exercise for >3 days per week), and income (<20th percentile) were obtained through a standardized questionnaire. The participants’ body mass indexes (BMIs) were calculated with height and weight, and the participants were categorized as follows: BMI  < 18.5, BMI = 18.5–22.9, BMI = 23.0–24.9, BMI = 25.0–29.9, and BMI  ≥ 30.0. Systolic and diastolic blood pressure and blood data, including glomerular filtration rate and high-density lipoprotein, low-density lipoprotein, and fasting glucose levels, were obtained after overnight fasting.

### 2.5. Statistical Analysis

The event incidence rate (IR) was calculated as the number of events per 1000 person-years. To investigate the risk of MI or IS in individuals diagnosed with uterine myomas, we analyzed the hazard ratios (HRs) of MI or IS by performing multivariate adjustments with confounders from a non-adjusted model to a fully adjusted model (Model 1: non-adjusted; Model 2: age, smoking, alcohol drinking, and regular exercise; Model 3: age, smoking, alcohol drinking, regular exercise, hypertension, DM, dyslipidemia, CKD, and BMI). The Student *t* test was used to compare continuous variables, and categorical variables were compared using the chi-square test. Cox regression analyses were performed to calculate the hazard ratio (HR) and 95% CI of each independent variable. Statistical significance was defined as *p* < 0.05. Statistical analyses were performed using SAS, version 9.4 (SAS Institute Inc., Cary, NC, USA) and R software, version 3.6.4 (R Foundation for Statistical Computing, Vienna, Austria).

### 2.6. Statement of Ethics

These data were restricted to those with access to the NHIS. We applied for access to the NHIS using a study protocol that was approved by the Institutional Review Board of the principal investigator’s affiliation and the NHIS (NHIS-2016-2-243). This study was approved by the Institutional Review Board of Seoul St. Mary’s Hospital (KIRB-0E513-001). Informed consent was not obtained because the data were anonymized and de-identified by the NHIS before analysis.

## 3. Results

### 3.1. Baseline Characteristics of the Study Population

The demographics of the 2,580,349 participants are shown in Table 1. A total of 58,812 women were diagnosed with uterine fibroids, of which 25,063 underwent surgery for leiomyoma. During the follow-up period, 196 of 58,812 patients with myomas were diagnosed with MI or IS.

The group of women with MI or IS consisted of older women (mean [SD] age, (29.75 [5.2] vs. 31.59 [5.4]), those with greater BMI (23.52% vs. 34.08%), more current smokers (5.92% vs. 9.52%), and more frequent consumers of alcoholic drinks (2.39% vs. 3.24%) than the group without MI or IS. Regarding comorbidities, women with MI or IS had a higher prevalence of diabetes mellitus and hypertension (all *p* < 0.001).

### 3.2. Risk of CVD with Uterine Myomas

Among the 58,812 patients with myomas in the 20–40-year age group who underwent health screening during the observation period, 115 (0.21%) experienced MI and 82 (0.15%) experienced stroke. In the group of 2,522,888 individuals without myoma, 3107 cases of MI (0.12%) and 2240 cases of stroke (0.09%) were reported. These findings indicate that the incidence of cardiovascular disease (CVD) events was higher in the group with uterine myomas.

In the case of MI, the hazard ratio (HR) was 1.59 (Table 2, Model 1,95% CI 1.32–1.91) in the myoma group compared to the non-myoma group, and this association was statistically significant even after adjusting for age, lifestyle, comorbidities, and BMI (Table 2, Model 3, HR 1.37, 95% CI 1.33–1.65). The risk of IS was 1.5-fold higher in the myoma group than in the non-myoma group; however, when adjusted for other factors, the elevated risk showed a trend that was not statistically significant.

When all CVD outcomes were analyzed, the myoma group showed a higher risk for CVD events even after adjusting for age, lifestyle, BMI, and comorbidities (Table 2, Model 3, HR 1.32, 95% CI 1.14–1.52). Furthermore, the IR analysis of overall CVD events based on uterine myoma status showed that the risk of IR in the myoma group was 0.63 compared to 0.39 in the non- myoma group, representing approximately a 1.58-fold higher rate of occurrence (Table 2).

Among patients with myomas, the history of surgical treatment was analyzed in relation to the overall cardiovascular disease (CVD) risk. In the fully adjusted model for all factors regarding the risk of overall CVD events, the group with a history of surgical intervention showed a lower CVD risk (1.34, 95% CI 1.11–1.62) compared to the non-surgical group (1.28, 95% CI 1.03–1.59) (Table 3).

## 4. Discussion

Our study included a large, ethnically homogeneous, and nationally representative cohort of young Korean women of reproductive age. The particularly large sample size is a unique strength of this study. We specifically calculated only new cases of uterine myomas over a relatively long follow-up period of nearly 10 years.

The data from this large population-based cohort indicate a potential association between uterine myomas and an increased risk of cardiovascular disease events, including myocardial infarction and ischemic stroke, in young women of reproductive age. The incidences of MI and IS were significantly higher in women diagnosed with myomas than in those without, even after adjusting for confounding factors such as age, lifestyle, comorbidities, and BMI. These results underline the importance of considering the systemic health implications in patients with uterine myomas.

The observed HRs of 1.59 for MI and 1.5 for IS in the myoma group highlighted a notable increase in cardiovascular risk. While the risk for MI remained statistically significant after adjustments, the elevated risk for IS showed a trend but was not statistically significant after full adjustment. This discrepancy may reflect the differences in the underlying mechanisms linking myomas to these two conditions.

Uterine leiomyomas represent a typical fibrotic disease characterized by the upregulation of extracellular matrix (ECM) proteins, particularly collagen 1A1, fibronectin, and versican [22]. Typically, fibroblasts are activated by chronic low-grade inflammatory signals related to obesity, infections, smoking, and menstrual history, leading to their differentiation into myofibroblasts [23,24]. Dysregulation of myofibroblast activity is implicated in pathological fibrosis and the growth of fibroids. Similarly, myocardial infarction (MI) also results in extensive remodeling of collagen and other ECM proteins, which occurs as part of the healing process following ischemic injury [25]. Macrophages play a crucial role in both conditions, contributing to tissue repair and the fibrotic process in MI, as well as modulating inflammation and fibrosis in uterine myomas [26]. The common observations of ECM remodeling, inflammatory responses, and the role of immune cells—particularly macrophages—suggest a link between these two diseases [27]. Moreover, hypertension is a known risk factor for MI, and several studies have demonstrated an association between hypertension and myomas, indicating the presence of shared risk factors for both conditions [16,27,28]. The increased demand for blood supply due to large or multiple myomas may lead to the occurrence of myocardial ischemia and anemia, which can contribute to an initial decline in cardiac pumping ability and, in the long term, increase myocardial contractility and oxygen demand, potentially exacerbating cardiovascular disease [29,30,31]. Additionally, cases involving conditions such as May–Thurner syndrome, where a uterine mass leads to compression of the iliac veins, resulting in paradoxical embolization and stroke in young women, suggest a potential link between the risk of stroke and uterine myomas [32,33,34]. However, the relationship between stroke and myomas may be influenced by additional, less well-understood factors that require further exploration.

Our analysis of overall CVD outcomes revealed an IR 1.58 times higher in the myoma group than in the non-myoma group. These findings underscore the need for enhanced cardiovascular risk assessment and monitoring of women with uterine myomas, particularly those with comorbid risk factors such as obesity, smoking, hypertension, and diabetes.

Interestingly, a subgroup analysis on the impact of surgical treatment of myoma on CVD risk showed a lower risk of CVD compared to the non-surgical treatment group. This suggests that surgical intervention may have additional benefits that reduce cardiovascular risk factors. According to the study by Kirschen GW et al., the removal of fibroids was associated with a slight reduction in systolic blood pressure but had no effect on diastolic blood pressure [20]. However, when analyzing MI and IS separately, as representative CVD conditions, no significant trend of risk reduction through surgical treatment was observed. Women undergoing surgery may represent a subset with more severe or symptomatic myomas, and further research is needed to explore the mechanisms by which the correction of symptoms through surgical treatment may reduce risk factors for cardiovascular disease.

These findings highlight the multifaceted nature of the relationship between myomas and CVD. The pathophysiological mechanisms underlying this association extend beyond localized uterine changes and involve systemic inflammation, vascular dysfunction, and hormonal influences [35,36]. Additionally, lifestyle factors, such as smoking and obesity, which were more prevalent among women with CVD events in this study, likely play a synergistic role in amplifying the cardiovascular risk in this population [37,38].

This study has several limitations. Because this was a retrospective cohort study, it was inherently observational, which made it difficult to establish causality. Although an association between uterine myomas and CVDs has been identified, whether this relationship is directly causal or mediated by shared factors remains unclear. Additionally, the study relied on insurance claims data, which means that the diagnostic accuracy was dependent on hospital records. Recent study has suggested that the genetics of the systemic vascular system can play a role in the onset of myomas and that genetic variations are associated with the size of myomas [39]. However, this study relies solely on disease codes for diagnosis, limiting the ability to perform analyses that reflect precise pathophysiological characteristics and individual differences. Mild cases of myomas or CVD may have been under-reported or missed. Lifestyle factors, such as smoking, alcohol consumption, and exercise, were based on self-reported data, which could have reduced the accuracy. Furthermore, while the impact of myomectomy on CVD was analyzed, it was challenging to ascertain the underlying reasons for surgical treatment.

However, this study underscores the need for integrated care approaches that address the gynecological and systemic health of women with myomas. Routine cardiovascular risk screening and proactive management of modifiable risk factors such as obesity and smoking should be considered in the clinical management of these patients. Furthermore, future research should aim to elucidate the biological mechanisms linking myomas to cardiovascular outcomes and explore strategies to mitigate these risks, particularly in women undergoing surgical treatments, such as myomectomy.

In conclusion, our findings indicate that myomas are not merely a localized gynecological condition but may have significant systemic implications for cardiovascular health. This study contributes to the growing body of evidence supporting the need for a more holistic approach to the management of myomas considering their potential impact on broader health outcomes.

## Figures and Tables

**Table 1:** Baseline characteristics of the study population.

Characteristics	Total, No. (%)	CVD, No. (%)		*p*-Value
		No	Yes	
	2,580,349	2,575,406	4943	
Age, mean ± SD	29.75 ± 5.23	29.74 ± 5.23	31.59 ± 5.37	<0.0001
<30	1,336,189 (51.76)	1,334,172 (51.79)	2017 (36.87)	<0.0001
30≤	1,245,511 (48.24)	1,242,058 (48.21)	3453 (63.13)	
Uterine fibroid				<0.0001
No	2,522,888 (97.72)	2,517,614(97.72)	5274 (96.42)	
Yes	58,812 (2.28)	58,616 (2.28)	196 (3.58)	
Myomectomy				<0.0001
No	2,556,637 (99.03)	2,551,253 (99.03)	5384 (98.43)	
Yes	25,063 (0.97)	24,977 (0.97)	86 (1.57)	
Diabetes mellitus	24,431 (0.95)	24,257 (0.94)	174 (3.18)	<0.0001
Hypertension	60,544 (2.35)	60,173 (2.34)	371 (6.78)	<0.0001
Dyslipidemia	96,646 (3.74)	96,256 (3.74)	390 (7.13)	<0.0001
CKD	71,245 (2.76)	71,082 (2.76)	163 (2.98)	0.3195
Smoking				<0.0001
Non	2,333,989 (90.41)	2,329,292 (90.41)	4697 (85.87)	
Past	94,660 (3.67)	94,408 (3.66)	252 (4.61)	
Current	153,051 (5.93)	152,530 (5.92)	521 (9.52)	
Alcohol drinking				<0.0001
Non	1,407,627 (54.52)	1,404,611 (54.52)	3016 (55.14)	
Mild	1,112,295 (43.08)	1,110,018 (43.09)	2277 (41.63)	
Heavy	61,778 (2.39)	61,601 (2.39)	177 (3.24)	
Regular exercise	246,691 (9.56)	246,084 (9.55)	607 (11.1)	0.0001
BMI (kg/m^2^)	21.36 ± 3.25	21.36 ± 3.25	22.31 ± 3.91	<0.0001
<18.5	381,218 (14.77)	380,587 (14.77)	631 (11.54)	<0.0001
<23	1,592,692 (61.69)	1,589,717 (61.71)	2975 (54.39)	
<25	301,165 (11.67)	300,394 (11.66)	771 (14.1)	
<30	245,700 (9.52)	244,887 (9.51)	813 (14.86)	
30≤	60,925 (2.36)	60,645 (2.35)	280 (5.12)	
Income (lower 20%)	561,729 (21.76)	560,353 (21.75)	1376 (25.16)	<0.0001
eGFR (mL/min/1.73 m^2^)	98.24 ± 41.22	98.25 ± 41.23	96.89 ± 37.21	0.0153
Fasting glucose (mg/dL)	88.2 ± 13.22	88.2 ± 13.19	91.44 ± 23.55	<0.0001
Systolic BP (mmHg)	111.25 ± 11.53	111.24 ± 11.52	114.04 ± 13.94	<0.0001
Diastolic BP (mmHg)	69.86 ± 8.52	69.85 ± 8.52	71.81 ± 10.03	<0.0001
Baseline HDL-C (mg/dL)	63.39 ± 27.55	63.39 ± 27.54	61.92 ± 30.12	<0.0001
Baseline LDL-C (mg/dL)	112.14 ± 302.12	112.14 ± 302.19	112.84 ± 270.37	0.8645

Data are expressed as the mean  ±  SD or *n* (%); BMI, body mass index; BP, blood pressure; CKD, chronic kidney disease; CVD, cardiovascular disease; eGFR, estimated glomerular filtration rate; HDL, high-density lipoprotein; LDL, low-density lipoprotein.

**Table 2:** Risk of myocardial infarction and stroke in patients with or without uterine fibroid.

	Fibroid	Total *n*	Event	IR Per 1000	Model 1	*p*-Value	Model 2	*p*-Value	Model 3	*p*-Value
MI	No	2,522,888	3107	0.23	1 (Ref.)	<0.0001	1 (Ref.)	0.0008	1 (Ref.)	0.0011
	Yes	58,812	115	0.37	1.58 (1.32, 1.91)		1.36 (1.14, 1.66)		1.36 (1.13, 1.64)	
Stroke	No	2,522,888	2240	0.17	1 (Ref.)	<0.0001	1 (Ref.)	0.0567	1 (Ref.)	0.0708
	Yes	58,812	82	0.26	1.57 (1.26, 1.96)		1.24 (0.99, 1.55)		1.26 (0.98, 1.53)	
CVD	No	2,522,888	5274	0.39	1 (Ref.)	<0.0001	1 (Ref.)	0.0001	1 (Ref.)	0.0002
	Yes	58,812	196	0.63	1.59 (1.38, 1.84)		1.33 (1.15, 1.53)		1.32 (1.14, 1.52)	

Model 1: Non-adjusted. Model 2: Age, smoking, drinking, regular exercise. Model 3: Age, smoking, drinking, regular exercise, DM, HP, DYS, CKD, and BMI. BMI, body mass index; CKD, chronic kidney disease; CVD, cardiovascular disease; DM, diabetes mellitus; MI, myocardial infarction.

**Table 3:** Risk of myocardial infarction and stroke based on the history of myomectomy in patients with uterine fibroids.

	Fibroid	*n*	Event	IR per 1000	Model 1	*p*-Value	Model 2	*p*-Value	Model 3	*p*-Value
MI	No	2,522,888	3107	0.23	1 (Ref.)	<0.0001	1 (Ref.)	0.0027	1 (Ref.)	0.003
	Yes − OP	33,749	68	0.38	1.62 (1.30, 2.10)		1.46 (1.15, 1.86)		1.462 (1.15, 1.86)	
	Yes + OP	25,063	47	0.35	1.50 (1.13, 2.00)		1.27 (0.95, 1.70)		1.248 (0.94, 1.67)	
Stroke	No	2,522,888	2240	0.17	1 (Ref.)	0.0002	1 (Ref.)	0.1392	1 (Ref.)	0.1788
	Yes − OP	33,749	43	0.24	1.45 (1.07, 1.95)		1.18 (0.87, 1.59)		1.18 (0.87, 1.59)	
	Yes + OP	25,063	39	0.29	1.74 (1.26, 2.38)		1.32 (0.96, 1.81)		1.28 (0.94, 1.76)	
CVD	No	2,522,888	5274	0.40	1 (Ref.)	<0.0001	1 (Ref.)	0.0005	1 (Ref.)	0.0008
	Yes − OP	33,749	110	0.62	1.57 (1.30, 1.90)		1.34 (1.11, 1.62)		1.34 (1.11, 1.62)	
	Yes + OP	25,063	86	0.64	1.62 (1.31, 2.01)		1.31 (1.06, 1.62)		1.28 (1.036, 1.59)	

Model 1: Non-adjusted. Model 2: Age, smoking, drinking, regular exercise. Model 3: Age, smoking, drinking, regular exercise, DM, HP, DYS, CKD, and BMI. BMI, body mass index; CKD, chronic kidney disease; CVD, cardiovascular disease; DM, diabetes mellitus; MI, myocardial infarction; OP, operation.

## Data Availability

The raw data supporting the conclusions of this article will be made available by the authors on request.

## References

[1] Giuliani E., As-Sanie S., Marsh E.E. (2020). Epidemiology and management of uterine fibroids. Int. J. Gynecol. Obstet..

[2] Stewart E.A., Cookson C.L., Gandolfo R.A., Schulze-Rath R. (2017). Epidemiology of uterine fibroids: A systematic review. BJOG Int. J. Obstet. Gynaecol..

[3] McWilliams M.M., Chennathukuzhi V.M. (2017). Recent advances in uterine fibroid etiology. Seminars in Reproductive Medicine.

[4] Lou Z., Huang Y., Li S., Luo Z., Li C., Chu K., Zhou J. (2023). Global, regional, and national time trends in incidence, prevalence, and years lived with disability for uterine fibroids, 1990–2019: An age-period-cohort analysis for the Global Burden of Disease 2019 study. BMC Public Health.

[5] Stewart E.A. (2001). Uterine fibroids. Lancet.

[6] Stewart E.A., Laughlin-Tommaso S.K., Catherino W.H., Lalitkumar S., Gupta D., Vollenhoven B. (2016). Uterine fibroids. Nat. Rev. Dis. Primers.

[7] Mitro S.D., Wise L.A., Waetjen L.E., Lee C., Zaritsky E., Harlow S.D., Solomon D.H., Thurston R.C., El Khoudary S.R., Santoro N. (2024). Hypertension, cardiovascular risk factors, and uterine fibroid diagnosis in midlife. JAMA Netw. Open.

[8] Brewster L.M., Haan Y., Van Montfrans G.A. (2022). Cardiometabolic risk and cardiovascular disease in young women with uterine fibroids. Cureus.

[9] Haan Y.C., Diemer F.S., Van Der Woude L., Van Montfrans G.A., Oehlers G.P., Brewster L.M. (2018). The risk of hypertension and cardiovascular disease in women with uterine fibroids. J. Clin. Hypertens..

[10] Laughlin-Tommaso S.K., Fuchs E.L., Wellons M.F., Lewis C.E., Calderon-Margalit R., Stewart E.A., Schreiner P.J. (2019). Uterine fibroids and the risk of cardiovascular disease in the Coronary Artery Risk Development in Young Adult Women Study. J. Women’s Health.

[11] Cohen-Solal A., Leclercq C., Deray G., Lasocki S., Zambrowski J.J., Mebazaa A., de Groote P., Damy T., Galinier M. (2014). Iron deficiency: An emerging therapeutic target in heart failure. Heart.

[12] Von Haehling S., Jankowska E.A., Van Veldhuisen D.J., Ponikowski P., Anker S.D. (2015). Iron deficiency and cardiovascular disease. Nat. Rev. Cardiol..

[13] Dubey P., Reddy S., Singh V., Yousif A., Dwivedi A.K. (2024). Association of heavy menstrual bleeding with cardiovascular disease in US female hospitalizations. BMC Med..

[14] Lee G., Choi S., Kim K., Yun J.M., Son J.S., Jeong S.M., Park S.M. (2018). Association between changes in hemoglobin concentration and cardiovascular risks and all-cause mortality among young women. J. Am. Heart Assoc..

[15] Chen Y., Xiong N., Xiao J., Huang X., Chen R., Ye S., Tan X. (2022). Association of uterine fibroids with increased blood pressure: A cross-sectional study and meta-analysis. Hypertens. Res..

[16] Boynton-Jarrett R., Rich-Edwards J., Malspeis S., Missmer S.A., Wright R. (2005). A prospective study of hypertension and risk of uterine leiomyomata. Am. J. Epidemiol..

[17] Haan Y.C., Oudman I., de Lange M.E., Timmermans A., Ankum W.M., van Montfrans G.A., Brewster L.M. (2015). Hypertension risk in Dutch women with symptomatic uterine fibroids. Am. J. Hypertens..

[18] Uimari O., Auvinen J., Jokelainen J., Puukka K., Ruokonen A., Järvelin M.R., Martikainen H. (2016). Uterine fibroids and cardiovascular risk. Hum. Reprod..

[19] Aksoy Y., Sivri N., Karaoz B., Sayin C., Yetkin E. (2014). Carotid intima-media thickness: A new marker of patients with uterine leiomyoma. Eur. J. Obstet. Gynecol. Reprod. Biol..

[20] Kirschen G.W., Yanek L., Borahay M. (2023). Relationship among surgical fibroid removal, blood pressure, and biomarkers of renin-angiotensin-aldosterone system activation. Reprod. Sci..

[21] Korean Statistical Office 2023 Mortality Statistics. https://kostat.go.kr/board.es?mid=a10301010000&bid=218&act=view&list_no=433106.

[22] Cardozo E.R., Foster R., Karmon A.E., Lee A.E., Gatune L.W., Rueda B.R., Styer A.K. (2018). MicroRNA 21a-5p overexpression impacts mediators of extracellular matrix formation in uterine leiomyoma. Reprod. Biol. Endocrinol..

[23] Wegienka G. (2012). Are uterine leiomyoma a consequence of a chronically inflammatory immune system?. Med. Hypotheses.

[24] Weiss G., Goldsmith L.T., Taylor R.N., Bellet D., Taylor H.S. (2009). Inflammation in reproductive disorders. Reprod. Sci..

[25] Mouton A.J., Rivera O.J., Lindsey M.L. (2018). Myocardial infarction remodeling that progresses to heart failure: A signaling misunderstanding. Am. J. Physiol. Heart Circ. Physiol..

[26] Ross R. (1999). Atherosclerosis—An inflammatory disease. N. Engl. J. Med..

[27] Faerstein E., Szklo M., Rosenshein N.B. (2001). Risk factors for uterine leiomyoma: A practice-based case-control study. II. Atherogenic risk factors and potential sources of uterine irritation. Am. J. Epidemiol..

[28] Silver M.A., Raghuvir R., Fedirko B., Elser D. (2005). Systemic hypertension among women with uterine leiomyomata: Potential final common pathways of target end-organ remodeling. J. Clin. Hypertens..

[29] Okoth K., Smith W.P., Thomas G.N., Nirantharakumar K., Adderley N.J. (2023). The association between menstrual cycle characteristics and cardiometabolic outcomes in later life: A retrospective matched cohort study of 704,743 women from the UK. BMC Med..

[30] Ponikowski P., Voors A.A., Anker S.D., Bueno H., Cleland J.G., Coats A.J., Falk V., González-Juanatey J.R., Harjola V.P., Jankowska E.A. (2016). 2016 ESC Guidelines for the diagnosis and treatment of acute and chronic heart failure. Kardiol. Pol. (Pol. Heart J.).

[31] Hsu H.N., Hsu F.C., Hung Y., Hsu P.C., Su K.M. (2024). Heart failure associated with giant uterine leiomyoma: A case report. Medicina.

[32] Schapiro L., Thorp J.M. (1996). Management of leiomyoma causing myocardial infarction. Eur. J. Obstet. Gynecol. Reprod. Biol..

[33] Higuchi E., Toi S., Shirai Y., Mizuno S., Onizuka H., Nagashima Y., Hashimoto K., Kitagawa K. (2019). Recurrent cerebral infarction due to benign uterine myoma. J. Stroke Cerebrovasc. Dis..

[34] Naito H., Naka H., Kanaya Y., Yamazaki Y., Tokinobu H. (2014). Two cases of acute ischemic stroke associated with iron deficiency anemia due to bleeding from uterine fibroids in middle-aged women. Intern. Med..

[35] Walker C.L., Stewart E.A. (2005). Uterine fibroids: The elephant in the room. Science.

[36] Lira-Silva E., Del Valle Mondragón L., Pérez-Torres I., Posadas-Sánchez R., Roldán Gómez F.J., Posadas-Romero C., Vargas-Barrón J., Pavón N. (2023). Possible implication of estrogenic compounds on heart disease in menopausal women. Biomed. Pharmacother..

[37] Burkman R.T. (1988). Obesity, stress, and smoking: Their role as cardiovascular risk factors in women. Am. J. Obstet. Gynecol..

[38] Rippe J.M. (2018). Lifestyle strategies for risk factor reduction, prevention, and treatment of cardiovascular disease. Am. J. Lifestyle Med..

[39] Inácio Â., Aguiar L., Rodrigues B., Pires P., Ferreira J., Bilhim T., Pisco J., Bicho M., Bicho M.C. (2024). Leiomyoma and the Importance of Genetic Variation on Genes Related to the Vasculature System—CβS, MTHFR, NOS3, CYBA, and ACE1. Eur. J. Obstet. Gynecol. Reprod. Biol..

